# Time Course of Recovery Following Resistance Exercise with Different Loading Magnitudes and Velocity Loss in the Set

**DOI:** 10.3390/sports7030059

**Published:** 2019-03-04

**Authors:** Fernando Pareja-Blanco, Antonio Villalba-Fernández, Pedro J. Cornejo-Daza, Juan Sánchez-Valdepeñas, Juan José González-Badillo

**Affiliations:** Physical Performance & Athletic Research Center, Faculty of Sports Science, Pablo de Olavide University, Ctra. Utrera km 1, 41013 Seville, Spain; antonvillacorre@gmail.com (A.V.-F.); pjcordaz@gmail.com (P.J.C.-D.); juan_valdemate@hotmail.com (J.S.-V.); jjgbadi@gmail.com (J.J.G.-B.)

**Keywords:** velocity-based training, strength training, full squat, running sprint, short-term recovery, vertical jump

## Abstract

The aim of this study was to compare the time course of recovery following four different resistance exercise protocols in terms of loading magnitude (60% vs. 80% 1RM—one-repetition maximum) and velocity loss in the set (20% vs. 40%). Seventeen males performed four different protocols in full squat exercise, which were as follows: (1) 60% 1RM with a velocity loss of 20% (60-20), (2) 60% 1RM with a velocity loss of 40% (60-40), (3) 80% 1RM with a velocity loss of 20% (80-20), and (4) 80% 1RM with a velocity loss of 40% (80-40). Movement velocity against the load that elicited a 1 m·s^−1^ velocity at baseline measurements (V_1_-load), countermovement jump (CMJ) height, and sprint time at 20 m (T20) were assessed at Pre, Post, 6 h-Post, 24 h-Post, and 48 h-Post. Impairments in V_1_-load were significantly higher for 60-40 than other protocols at Post (*p* < 0.05). The 60-20 and 80-40 protocols exhibited significant performance impairments for V_1_-load at 6 h-Post and 24 h-Post, respectively (*p* < 0.05). CMJ height remained decreased for 60-20 and 60-40 until 24 h-Post (*p* < 0.001–0.05). Regarding T20, the 80-40 protocol resulted in higher performance than 60-40 at 24 h-Post and the 80-20 protocol induced a greater performance than 60-40 protocol at 48 h-Post (*p* < 0.05). A higher velocity loss during the set (40%) and a lower relative load (60% 1RM) resulted in greater fatigue and slower rate of recovery than lower velocity loss (20%) and higher relative load (80% 1RM).

## 1. Introduction

Among the main resistance exercise variables that can be manipulated to configure mechanical stimulus, it appears that exercise intensity and volume are among the most critical factors in determining the type and extent of the resulting neuromuscular adaptations [1,2]. The interaction between these two training variables produces what is termed ‘level of effort’, which is defined as the actual number of repetitions performed in a set in relation to the maximum number that can be completed [3]. The indicators that have traditionally been used as references for quantifying and prescribing the resistance training (RT) load (one-repetition maximum, “1RM” and maximum number of repetitions test, “nRM”) have potential limitations, such as daily changes in the actual 1RM, which mean that the current 1RM may not correspond with that measured on previous days or weeks. Therefore, it cannot be ensured that the relative loads (%1RM) being used in each particular training session truly represent the intended ones. Another disadvantage of the nRM method is that the maximal number of repetitions that can be completed against a given relative load indicates a large variability between individuals [4,5]. Therefore, 10RM does not necessarily constitute the same load (%1RM) for every participant. These limitations led researchers and coaches to seek a solution that allows a better definition and quantification of the level of effort involved during RT. In this regard, a new approach, known as velocity-based training (VBT), has emerged, using movement velocity for objectively quantifying and dosing RT programs [3,6,7].

A pioneering study found an extremely close relationship between %1RM and movement velocity (R² = 0.98) in the bench press exercise [6]. Additional research has also observed close relationships between loading magnitude and movement velocity in other exercises (prone bench pull, full squat, half-squat, and pull-up) [8,9,10,11]. The extremely close relationship between %1RM and movement velocity allows researchers and coaches to determine with considerable accuracy what %1RM is being used as soon as the first repetition of a set is performed with maximal voluntary velocity [6,9]. Such findings open up the possibility of monitoring, in real time, the actual load (%1RM) being used, by measuring velocity during training. This allows determination of whether the proposed load (kg) truly represents the %1RM that was intended for each session [6]. Even more relevant is the fact that strength and conditioning coaches can detect the changes in strength that occur during the course of a training program, without the need to perform the often demanding, time-consuming and interfering 1RM or nRM assessments every few training sessions. 

Traditionally, training volume is prescribed using a fixed number of repetitions in each exercise set for all participants. In this regard, a recent work has reported that other variables such as movement velocity or time under tension, rather than the nRM are critical variables in assessment of training volume [12]. Moreover, the nRM that can be completed against a given relative load (%1RM) shows large inter-individual variability [4,5]. Hence, if two athletes perform the same number of repetitions per set against a given relative load, it is possible that they are exerting a different level of effort (i.e., the number of repetitions left in reserve in each set may vary considerably between individuals). A recent study has shown a close relationship between the percentage of velocity loss incurred in a set and the percentage of completed repetitions with respect to the maximum number of repetitions that can be performed (R² = 0.96) [4]. In this way, it is possible to determine with considerable precision the percentage of repetitions that has been completed from the velocity loss incurred in the set [4]. In the squat exercise, a velocity loss of 40%–50% in the set means that the set is conducted to, or very close to, muscle failure, whereas a velocity loss of 20% means that the athlete has performed ∼50% of the possible repetitions [3,13,14]. These findings support the validity of using velocity loss as a variable to objectively quantify the level of effort achieved during the set. Therefore, rather than performing a specific number of repetitions, it seems more appropriate to end each training set as soon as a certain level of neuromuscular fatigue is detected.

Resistance exercise can result in acute muscle fatigue that may continue for several hours to days following a workout. Several studies have shown that, among other factors, the time needed for recovery significantly increases as the repetition number approaches failure [13,14,15,16]. In order to compare the time course of recovery following each workout, vertical countermovement jump (CMJ) height and movement velocity against the load that elicited a 1 m·s^−1^ mean propulsive velocity (V_1_-load) were assessed at Pre, Post, 6 h-Post, 24 h-Post, and 48 h-Post [13,14,15,16]. These previous studies have shown reductions in the ability to rapidly apply force for up to 48 h following resistance exercise to failure against 70%, 75%, and 80% 1RM [13,14,15,16]. However, these studies prescribed the same number of repetitions for all participants with the same %1RM [13,14,15,16]. Because the maximal number of repetitions against a given %1RM shows great variability [4,5], it is possible that the level of effort induced for each participant was different. Moreover, it is possible that the level of fatigue, and consequently the time course of recovery, are different when different %1RM are employed [17]. However, to our knowledge, no study has analyzed the time course of recovery to resistance protocols with the same velocity loss induced in the set but with different relative loads. In light of these considerations, a more detailed knowledge of the short-term recovery from different relative intensities and percentages of velocity loss incurred during the set will enable strength and conditioning coaches to objectively establish the time of recovery that will allow athletes to attain greater neuromuscular performance in an upcoming competition event or the next workout. Therefore, the aim of this study was to analyze the time course of recovery following four different resistance exercise protocols (REPs) in terms of loading magnitude (60% and 80% 1RM) and velocity loss required (20% vs. 40%) in full squat (SQ) exercise. Several assessment time points up to 48 h post-exercise were established to evaluate the mechanical response to an acute REP using a VBT approach, which means prescribing training in terms of two variables: (1) first (usually fastest) repetition’s mean velocity, which is intrinsically related to loading magnitude [6], and (2) the maximum percentage of velocity loss allowed in each set [3]. 

## 2. Material and Methods

### 2.1. Experimental Design

Following familiarization all participants undertook four randomized resistance exercise protocols (REPs), performed 14 days apart in separate trials. Two different relative loads (60% vs. 80% 1RM) and two different magnitudes of velocity loss during the set (20% vs. 40%) were used. The same number of exercise sets (3) and inter-set rest duration (4 min) were used in all REPs. The experimental design thus comprised four REPs, which were as follows: (1) 60% 1RM with a velocity loss in the set of 20% (60-20), (2) 60% 1RM with a velocity loss in the set of 40% (60-40), (3) 80% 1RM with a velocity loss in the set of 20% (80-20), and (4) 80% 1RM with a velocity loss in the set of 40% (80-40). In each REP, as soon as the corresponding target velocity loss limit was exceeded, the set was terminated.

**Figure 1:**
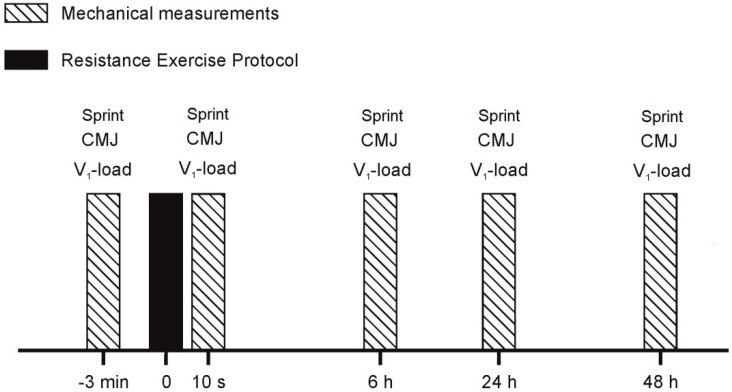
Representation of mechanical measurements at different time points to analyze the time course of recovery following exercise.

In order to compare the mechanical response, as well as the time course of recovery following each protocol analyzed, participants underwent a battery of measurements at different time points: pre-exercise (Pre), post-exercise (Post), 6 h-Post, 24 h-Post, and 48 h-Post. Movement velocity against the load that elicited a 1 m·s^−1^ mean propulsive velocity at baseline measurements (V_1_-load), vertical countermovement jump (CMJ) height, and running sprint time in 20 m (T20) were assessed at Pre, Post, 6 h-Post, 24 h-Post, and 48 h-Post (Figure 1). These mechanical measurements have been described in detail elsewhere [3]. The V_1_-load was chosen because it represents a sufficiently moderate loading intensity (~60% 1RM in SQ) [9] to allow ready detection of the effect of fatigue on movement velocity and quick to establish as part of the warm-up [3]. 

Participants refrained from any strenuous physical activity for at least 4 days before each REP trial. All REPs were performed at the same time of the day for each participant and under controlled environmental conditions (20–22 °C and 55%–65% humidity) in a research laboratory. Participants underwent four familiarization sessions two weeks before the start of the first trial. These sessions were supervised by researchers, and attention was paid to ensuring proper exercise lifting techniques were used and to providing detailed instruction on specific testing procedures. 

### 2.2. Participants

Seventeen men (age 23.6 ± 3.6 yr., height 1.80 ± 0.10 m, body mass 76.2 ± 10.9 kg) volunteered to participate in this study. Participants were physically active sports science students with RT experience ranging from 2.8 ± 1.1 yr. (2.1 ± 0.6 sessions per week), but they were not strength-trained athletes. Their initial 1RM was 111.4 ± 25.2 kg for the full squat (SQ) exercise. After being informed about the experimental procedures and the potential risks of the investigation, the participants gave their written informed consent to participate. No physical limitations, health problems, or musculoskeletal injuries that could affect testing were found after a medical examination. The study was approved by the Research Ethics Committee of Pablo de Olavide University and was conducted in accordance with the Declaration of Helsinki.

### 2.3. Measures

A Smith machine with no counterweight mechanism (Multipower Fitness Line, Peroga, Murcia, Spain) was used for all sessions. The SQ was performed with participants starting from the upright position with the knees and hips fully extended, feet approximately shoulder-width apart, and the barbell resting across the back at the level of the acromion. Each participant descended at a controlled pace (~0.50 m·s^−1^) until the tops of the thighs were below the horizontal plane, then immediately reversed motion and ascended back to the upright position. Participants were required to always execute the concentric phase at maximal intended velocity. This execution technique was carefully reproduced in all REPs performed in the study. All barbell repetitions were recorded with a linear velocity transducer (T-Force System, Ergotech, Murcia, Spain). The reliability of this setup has been documented elsewhere [3]. The velocity measures obtained in the present study correspond to the mean velocity of the propulsive phase (MPV) of each repetition. The propulsive phase was defined as that portion of the concentric phase during which barbell acceleration was greater than acceleration due to gravity [18].

### 2.4. Resistance Exercise Protocol

Figure 1 shows a detailed description of the protocol carried out. All REPs were performed in the morning (10 AM). The warm-up consisted of: 5 min jogging at a self-selected easy pace, four 20 m running accelerations and 10 m running all-out. Then, two 20 m running sprints separated by 3 min were performed and the best time was taken as the pre-exercise reference value (T20). A standing start with the lead-off foot placed 1 m before the first timing gate was used. Sprint times were measured using photocells (Witty, Microgate, Bolzano, Italy). Following the sprint test, two sets of 10 squats with no external load (i.e., own body mass), and five CMJs of increasing intensity were performed. Then, three maximal CMJs separated by 20 s rest periods were performed and the mean jump height was taken as the pre-exercise reference value. CMJ height was determined using an infrared timing system (OptojumpNext, Microgate, Bolzano, Italy). During the CMJ, the participant was instructed to rest his hands on his hips. All participants were instructed to land in an upright position and to bend the knees after landing. For the determination of the V_1_-load in SQ, 3 sets of 6, 4 and 3 repetitions, respectively (2 min inter-set rests) with increasing loads up to each participant’s V_1_-load were performed. The mean velocity of the 3 maximal intended repetitions with the V_1_-load was registered as the pre-exercise reference value for this variable, determined with a precision of ±0.05 m·s^−1^. Finally, the external load was adjusted to the intensity scheduled. Relative loads were determined from the load–velocity relationship since it has recently been shown that there is a very close relationship (R² = 0.95–0.98) between %1RM and MPV for the SQ exercise [9]. Thus, a target MPV to be attained in the first (usually the fastest) repetition of the first training set in each protocol was used as an estimation of %1RM, as follows: 1.00 m·s^−1^ for 60% 1RM and 0.68 m·s^−1^ for 80% 1RM. Subsequently, 3 sets separated by 4 min rest were performed using the designated protocol. Immediately after completing the final repetition of the third set (the load was changed in 10 s with the help of trained spotters), participants again performed 3 repetitions with the V_1_-load. Furthermore, 20 s after the SQ exercise, another 3 maximal CMJs, separated by 10 s rests, were performed. In addition, 1 min after the SQ exercise, a 20 m running sprint test was carried out. The V_1_-load, CMJ, and running sprint values were obtained as acute post-exercise measures. Strong verbal encouragement and velocity feedback were provided in each repetition throughout all exercise sets. 

At 4 PM in the evening (6 h-Post), and at 10 AM on the following two days (24 h-Post and 48 h-Post), the V_1_-load, CMJ, and running sprint measurements were repeated, as described above, in order to assess the time course of recovery following each specific protocol.

### 2.5. Measurements of Performance Impairments

Four different methods were used to quantify the extent of fatigue induced by each protocol. The first method analyzed the decline in repetition velocity during the three consecutive exercise sets and was calculated as the percent loss in MPV from the fastest to the slowest repetition of each set and averaged over the three sets. The second method examined the pre-to-post exercise change in velocity attained against the V_1_-load. The third and the fourth methods analyzed the change in CMJ height and T20 pre-post exercise. Test–retest reliability measured by the coefficient of variation (CV) was 1.8%, 0.8%, and 4.5% for CMJ, T20, and V_1_-load, respectively. The intraclass correlation coefficient (ICC) values were 0.993 (95% confidence interval, CI: 0.985–0.997), 0.984 (95%CI: 0.956–0.994), and 0.966 (95%CI: 0.880–0.994) for CMJ, T20, and V_1_-load, respectively.

### 2.6. Statistical Analyses

Values are reported as mean ± standard deviation (SD). Test–retest absolute reliability was measured by the standard error of measurement (SEM) which was expressed in relative terms through CV, whereas relative reliability was assessed by the ICC (95%CI) calculated with the one-way random effects model. The SEM was calculated as the root mean square of total mean square intra-subject. Statistical significance was established at P ≤ 0.05. At Pre, all data were normally distributed as determined by the Shapiro–Wilk test of normality. A factorial ANOVA with repeated measures with Bonferroni adjustment was used. Statistical analyses were performed using SPSS version 18.0 (SPSS Inc., Chicago, IL, USA).

## 3. Results

### 3.1. Descriptive Characteristics of the Resistance Exercise Protocols

Characteristics of each REP are reported in Table 1 in terms of repetitions performed per set (reps) and actual repetition velocities. The fastest repetition did not differ from the expected target velocities corresponding to each %1RM. Therefore, the highest velocity during each REP (Fastest-V) was higher for 60-20 and 60-40 than for 80-20 and 80-40 protocols. Relative loss of velocity within the set (MeanLoss-V) matched the aforementioned expected target velocity losses. Hence, both 60-20 and 80-20 achieved significantly lower MeanLoss-V than 60-40 and 80-40 protocols (Table 1). Both the slowest velocity measured in the three sets (Slowest-V) and the mean velocity during the training session (Mean-V) were lower in 80-40 compared to the other REPs (Table 1). In addition, the 80-20 protocol achieved lower Slowest-V and Mean-V than the 60-20 and 60-40 REPs. Lastly, 60-40 also achieved lower values in these variables than the 60-20 protocol. The 60-40 protocol resulted in more completed repetitions per set (reps) than the other REPs (Table 1). In addition, both 60-20 and 80-40 performed more reps than 80-20, without significant differences between the 60-20 and 80-40 protocols (Table 1). 

### 3.2. Time Course of Recovery

No significant differences between REPs were found at Pre for any of the variables analyzed (Table 2). Table 3 shows the changes in the mechanical variables analyzed following each REP. All REPs showed a significant decrease in performance at Post, except 80-40 in T20. Impairments in V_1_-load were significantly higher for 60-40 than other REPs at Post. In addition, 60-20 exhibited significant performance impairments for V_1_-load at 6h-Post, and the 80-40 protocol remained decreased until 24 h-Post. For CMJ height, 60-40 resulted in significantly greater performance impairment than 80-20 and 80-40 at Post. CMJ height remained decreased for both 60-20 and 60-40 until 24 h-Post (Table 3). Regarding T20, the 80-40 protocol resulted in higher performance than 60-40 at 24 h-Post and the 80-20 protocol induced a greater performance than the 60-40 protocol at 48 h-Post.

## 4. Discussion

To our knowledge, this is the first study that has analyzed the time course of recovery from different levels of effort (actual number of repetitions performed in relation to the maximum possible number) in each exercise set using a VBT approach; it followed distinct velocity losses during the set (20% vs. 40%). In addition, we compared these magnitudes of velocity loss for two different relative loads (60% vs. 80%1RM). The time course of recovery up to 48 h post-exercise following four distinct REP, 60-20 vs. 60-40 vs. 80-20 vs. 80-40, was compared. The fact that movement velocity was measured and recorded for every repetition allowed us to isolate the effect of the variables of interest; in this case, velocity loss, relative load, and the interaction between both. While it may seem obvious that halving the maximum possible number of repetitions per set induces greater fatigue, the important findings of this study are the implications this may have for the subsequent recovery. Interestingly, both 60% 1RM protocols (60-20 and 60-40) did not fully return to pre-exercise values at 48 h-Post for any variable analyzed (V1-load, CMJ, and T20). In this regard, the 80-20 protocol even exhibited greater sprint performance at 48 h-Post compared to Pre-exercise. Therefore, a higher velocity loss during the set (40%) and a lower relative load (60% 1RM) resulted in greater fatigue and slower rate of recovery than lower velocity loss (20%) and higher relative load (80% 1RM). 

We verified that the target velocities corresponding to 60% and 80% 1RM (1.00 and 0.68 m·s^−1^, respectively) and the expected velocity losses (20% and 40%) were met (Table 1). Hence, this study provides very accurate information about the actual effort performed, as it can be observed in Table 1. This resulted in a mechanical stimulus comprised of slower movement velocities for the 80-40 protocol compared to the other protocols, whereas the 60-20 REP induced faster velocities. In addition, the 60% 1RM protocols allowed athletes to perform more repetitions to induce the same magnitude of velocity loss than the 80% 1RM protocols. It is worth noticing that the 60-40 protocol resulted in significantly greater reductions in V_1_-load than the other protocols (Table 3). Moreover, the 60-20 protocol exhibited significant performance impairments for V_1_-load at 6h-Post, while the 80-40 protocol remained decreased until 24 h-Post and only the 80-20 showed fully restored baseline values (100%) at 48 h-Post. These results were even more evident for jumping ability, since the CMJ height remained decreased for both the 60-20 and 60-40 until 24 h-Post. Likewise, the 60-40 protocol induced higher sprint performance reduction than the 80-40 and 80-20 protocols at 24 h-Post and at 48 h-Post, respectively (Table 3). Taken together, it seems that the ability to develop force with the lower limbs may be exercise-dependent (squat, CMJ, running sprint). Sprint performance required shorter restoration time compared to the other exercises (squat and CMJ). Supporting this, a previous study showed that sprint performance requires a much shorter restoration time compared to CMJ performance [19]. Several previous studies also showed that the CMJ test offers superior sensitivity to altered neuromuscular function than other jump and sprint tests [19,20,21]. Additionally, physical performance may be considerably compromised up to 48 h following resistance exercise to failure, as indicated especially in those REPs that were characterized by a large number of repetitions and moderate intensities (60-40 protocol). This information is relevant because it provides meaningful feedback to coaches and athletes about the time course recovery induced by specific REP in relation to the resulting deterioration in physical performance (sprint, jump, and squat performance, Table 3). 

As expected, our results indicated that for a given %1RM, a higher magnitude of velocity loss in the set resulted in greater impairment of neuromuscular performance and slower post-exercise recovery. In accordance with these results, previous studies have also shown reductions in the ability to rapidly apply force for up to 48 h following resistance exercise to failure [13,14,15]. However, these studies prescribed the same number of repetitions for all participants with the same %1RM [13,15], which may induce a different level of effort for each participant, whereas we employed a VBT approach. Additionally, a recent paper analyzed the effects of 20% vs. 40% of velocity loss during the set on muscle phenotype [7]. It was found that a 40% velocity loss maximized the hypertrophic response along with a fast-to-slow shift in muscle phenotype. However, a velocity loss of 20% prevented this reduction in the fastest IIX fiber-type pool and resulted in similar or even superior strength gains [7]. Whether heavier or lighter loads induce greater fatigue has recently been an issue [22]. Some authors suggest that repetitive lifting of heavy loads would recruit more motor units at higher firing rates than a lighter load [23] and would result in greater fatigue due to reduced availability of non-fatigued motor units to recruit [17,24]. On the other hand, other authors have suggested that moderate loads leading to failure allow higher levels of mechanical work, as well as metabolic and hormonal stress, which would induce greater levels of fatigue [3,25]. Our findings suggest that, for the same magnitude of velocity loss incurred in the set, a greater degree of fatigue and slower recovery was experienced as loads decreased. This finding supports a recent paper that showed when the same percentage of velocity loss is incurred in the set, lower loads result in higher acute mechanical fatigue and lactate concentrations [26]. 

Our results suggest that a higher velocity loss during the set (40%) and a lower relative load (60% 1RM) resulted in greater fatigue and slower rate of neuromuscular recovery than lower velocity loss (20%) and higher relative load (80% 1RM). However, our findings are limited to the specific population analyzed (not strength-trained athletes), since a recent study has shown that the same relative stimulus induces in novice athletes a slower post-exercise recovery and higher muscle damage compared to experienced athletes [16]. As the main limitation of this study, we must acknowledge that there is no control group, therefore, the fatigue induced by the different time-point measures is unknown. Taken together, our findings show that both intensity and level of effort are two key variables to take into account when a training program is configured. The training intensity should be prescribed taking as a reference the velocity of the first (fastest) repetition and the level of effort prescribed as the percentage of velocity loss in the set. The quantification of these two variables may provide objective information about the degree of fatigue induced by the resistance training and the necessary time periods for the recovery of neuromuscular function. Additionally, recovery seems to be exercise-dependent. This methodology allows adjustments to be made to the training load at any time, resulting in better individualized training, especially in those sports where the performance goal is mainly focused on developing specific neuromuscular adaptations while attempting to prevent excessive fatigue that may interfere with other components of training. 

## Figures and Tables

**Table 1:** Descriptive characteristics of each resistance exercise protocol.

Intra-Session Variables	60-20	60-40	80-20	80-40
Fastest-V (m·s^−1^)	0.99 ± 0.04 ^82,84^	1.00 ± 0.05 ^82,84^	0.69 ± 0.03	0.70 ± 0.03
MeanLoss-V (%)	22.7 ± 2.5 ^64,84^	41.5 ± 3.1	23.5 ± 4.1 ^64,84^	43.6 ± 3.7
Slowest-V (m·s^−1^)	0.69 ± 0.05 ^64,82,84^	0.51 ± 0.06 ^82,84^	0.44 ± 0.06 ^84^	0.34 ± 0.03
Mean-V (m·s^−1^)	0.85 ± 0.04 ^64,82,84^	0.77 ± 0.07 ^82,84^	0.57 ± 0.04 ^84^	0.54 ± 0.03
Reps (n)	7.1 ± 2.1 ^64,82^	12.0 ± 5.1 ^82,84^	3.2 ± 1.1 ^84^	5.4 ± 2.8

Data are mean ± SD, n = 17. 60-20: protocol against 60% 1RM with a velocity loss in the set of 20%; 60-40: protocol against 60% 1RM with a velocity loss in the set of 40% (60-40); 80-20: protocol against 80% 1RM with a velocity loss in the set of 20%; 80-40: protocol against 80% 1RM with a velocity loss in the set of 40% (80-40); Fastest-V: highest velocity measured in the three sets; MeanLoss-V: mean percent loss in velocity from the fastest to the slowest repetition over the three sets; Slowest-V: lowest velocity measured in the three sets; Mean-V: mean velocity of all repetitions during the three sets; Reps: repetitions performed in each set. Velocities correspond to the mean concentric propulsive velocity of each repetition. Statistically significant differences with 60-40 protocol: ^64^ *p* < 0.05. Statistically significant differences with 80-20 protocol: ^82^ *p* < 0.05. Statistically significant differences with 80-40 protocol: ^84^ *p* < 0.05.

**Table 2:** Baseline values in every test before each resistance exercise protocol.

REP	T20 (s)	CMJ (cm)	V_1_-load (m·s^−1^)
60-20	2.99 ± 0.16	42.0 ± 6.2	1.01 ± 0.02
60-40	3.00 ± 0.12	42.7 ± 6.1	0.99 ± 0.04
80-20	3.00 ± 0.14	43.1 ± 3.8	0.98 ± 0.03
80-40	3.00 ± 0.14	43.3 ± 3.9	1.00 ± 0.04

Data are mean ± SD, n = 17. REP: resistance exercise protocol; 60-20: protocol against 60% 1RM with a velocity loss in the set of 20%; 60-40: protocol against 60% 1RM with a velocity loss in the set of 40% (60-40); 80-20: protocol against 80% 1RM with a velocity loss in the set of 20%; 80-40: protocol against 80% 1RM with a velocity loss in the set of 40% (80-40); T20: 20 m running sprint time; CMJ: countermovement jump; V_1_-load: velocity attained against the load that elicits a 1 m·s^−1^ in the pre-exercise.

**Table 3:** Comparison of changes in mechanical indicators of fatigue following each resistance exercise protocol.

**T20 (%)**				
**REP**	**Post**	**6 h-Post**	**24 h-Post**	**48 h-Post**
60-20	94.0 ± 4.6 *	99.1 ± 2.2	98.2 ± 2.8	98.0 ± 2.2^82^
60-40	90.9 ± 7.9 *	98.3 ± 4.1	97.1 ± 3.0	97.8 ± 4.3
80-20	96.0 ± 2.2 *	99.6 ± 3.4	99.5 ± 1.8	101.1 ± 2.0
80-40	97.6 ± 2.4	99.6 ± 2.2	100.6 ± 2.5 ^64^	100.3 ± 2.8
**CMJ (%)**				
**REP**	**Post**	**6 h-Post**	**24 h-Post**	**48 h-Post**
60-20	75.4 ± 1.9 **	92.2 ± 1.6 *	93.1 ± 2.1 *	95.2 ± 2.1
60-40	67.3 ± 2.6 **	91.9 ± 2.3 *	92.9 ± 2.0 *	93.4 ± 2.1
80-20	78.4 ± 1.8 **^64^	95.5 ± 1.9	95.5 ± 1.5	100.6 ± 1.1
80-40	76.7 ± 1.4 ** ^64^	96.5 ± 1.4	96.4 ± 1.7	99.9 ± 1.8
**V_1_-load (%)**				
**REP**	**Post**	**6 h-Post**	**24 h-Post**	**48 h-Post**
60-20	81.0 ± 8.0 ** ^64^	93.1 ± 7.7 *	92.2 ± 11.8	95.5 ± 10.5
60-40	67.4 ± 10.1 **	95.6 ± 12.2	92.6 ± 10.5	93.0 ± 13.9
80-20	78.7 ± 7.0 ** ^64^	95.9 ± 7.6	95.9 ± 6.1	100.8 ± 4.5
80-40	77.2 ± 8.8 ** ^64^	93.2 ± 8.1 *	89.2 ± 9.9 *	95.9 ± 9.3

Data are mean ± SD, n = 17. Values are expressed as percentage of initial (Pre) measures. REP: resistance exercise protocol; 60-20: protocol against 60% 1RM with a velocity loss in the set of 20%; 60-40: protocol against 60% 1RM with a velocity loss in the set of 40% (60-40); 80-20: protocol against 80% 1RM with a velocity loss in the set of 20%; 80-40: protocol against 80% 1RM with a velocity loss in the set of 40% (80-40); T20: 20 m running sprint time; CMJ: countermovement jump; V_1_-load: velocity attained against the load that elicits a 1 m·s^−1^ in the pre-exercise. Statistically significant differences with Pre at the corresponding time point: * *p* < 0.05, ** *p* < 0.001. Statistically significant differences with 60-40 protocol: ^64^ *p* < 0.05. Statistically significant differences with 80-20 protocol: ^82^ *p* < 0.05.
